# A Novel Bioreactor System Capable of Simulating the *In Vivo* Conditions of Synovial Joints

**DOI:** 10.1089/ten.tec.2020.0161

**Published:** 2020-12-16

**Authors:** Adel Tekari, Rainer J. Egli, Veit Schmid, Joern Justiz, Reto Luginbuehl

**Affiliations:** ^1^ Group for Bone Biology and Orthopaedic Research, Department for Biomedical Research, University of Bern, Bern, Switzerland.; ^2^ Graduate School for Cellular and Biomedical Sciences, University of Bern, Bern, Switzerland.; ^3^ Laboratory of Molecular and Cellular Screening Processes, Centre of Biotechnology of Sfax, University of Sfax, Sfax, Tunisia.; ^4^ RMS Foundation, Bettlach, Switzerland.; ^5^ Department of Diagnostic, Interventional and Pediatric Radiology, Inselspital, Bern University Hospital, University of Bern, Bern, Switzerland.; ^6^ Institute for Human-Centered Engineering (HuCE) BME Lab, Bern University of Applied Sciences, Biel, Switzerland.; ^7^ Blaser Swisslube AG, Hasle-Ruegsau, Switzerland.

**Keywords:** cartilage, bioreactor, tissue engineering, biomaterials, biomechanics

## Abstract

Any significant *in vitro* evaluation of cartilage tissue engineering and cartilage repair strategies has to be performed under the harsh conditions encountered *in vivo* within synovial joints. To this end, we have developed a novel automated physiological robot reactor system (PRRS) that is capable of recapitulating complex physiological motions and load patterns within an environment similar to that found in the human knee. The PRRS consists of a mechanical stimulation unit (MSU) and an automatic sample changer (ASC) within an environment control box in which the humidity, temperature, and gas composition are tightly regulated. The MSU has three linear (orthogonal) axes and one rotational degree of freedom (around the z-axis). The ASC provides space for up to 24 samples, which can be allocated to individual stimulation patterns. Cell-seeded scaffolds and *ex vivo* tissue culture systems were established to demonstrate the applicability of the PRRS to the investigation of the effect of load and environmental conditions on engineering and maintenance of articular cartilage *in vitro*. The bioreactor is a flexible system that has the potential to be applied for culturing connective tissues other than cartilage, such as bone and intervertebral disc tissue, even though the mechanical and environmental parameters are very different.

**Impact statement:**

The success of engineered cartilage tissues depends on the biological/biochemical stimulations parameters, which should be as close as possible to the conditions observed *in vivo*. The design of bioreactors should be, therefore, inspired from the *in vivo* conditions, rather than the application of one or two degree of freedom loading cycles.

## Introduction

Articular cartilage is a bradytroph and avascular tissue that is exposed to mechanical forces *in vivo*, which is essential to the development and maintenance of the structure and function of articular cartilage.^1^ Once damaged, the cartilage has only a limited potential for self-repair.^2^ Therefore, repair strategies using cells and/or tissue substitutes are required with the aim of restoring the function of cartilage.

*In vitro* evaluation of engineered or regenerated cartilage is of increasing importance, in particular, since regulatory requirements for market approval have tightened considerably over the past decade for tissue-engineered medical products.^3^ Accordingly, many bioreactor systems have been developed for cartilage tissue engineering. One of the aims has been the *in vitro* reproduction and study of the effect of mechanical stresses as experienced *in vivo* by cartilage. Most bioreactor systems, however, are very limited in their capability to accomplish mechanical loading, for example, by having implemented uniaxial compression only,^4–13^ or by using hydrostatic pressure in a closed environment.^14–21^ Only very few, more advanced bioreactors are able to apply mechanical stimulation in more than one degree of freedom, such as shear force as a second degree of freedom in addition to compression.^22–30^ Regardless of the advances that have been made, the mechanical stimulation provided by most reactor systems, however, fails to mimic physiological stresses and is limited to a few basic motion patterns. Moreover, most of them lack ambient control, in other words it is not possible to mimic the hypoxic environment as encountered *in vivo* within the synovial joints.^31^ Therefore, our goal was to design and to test an automated physiological robot reactor system (PRRS), which addresses these shortcomings. The following requirements were defined: (1) The mechanical stimulation unit (MSU) must be able to replicate loads as experienced within a knee joint; and (2) the hypoxic *in vivo* conditions of a synovial joint should be mimicked by an environmental control unit (ECB). Further, (3) the possibility of applying individual loading protocols to a series of samples within the same experiment was defined as the third requirement. Here, we discuss the setup of the bioreactor and demonstrate the capability of the PRRS with experiments using *in vitro* tissue engineering cultures and *ex vivo* articular cartilage. The effect on formation and maintenance of articular cartilage as a function of load was investigated in this study.

## Methods

### Design of PRRS

**FIG. 1.**
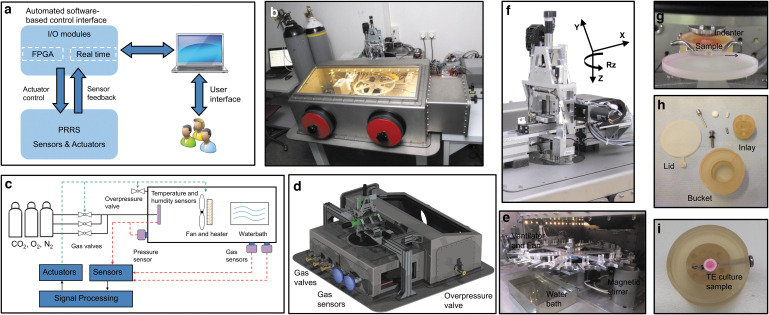
Design and assembly of components of the PRRS. The PRRS is connected to a personal computer with a user-friendly interface to control the device with I/O modules via actuators and sensors **(a)** and is connected to gas bottles to allow for regulation of the climate **(b–d)**. The main parts of the PRRS include an MSU **(f)** with four degree of freedom (three linear axes [x, y, and z] and the rotational axis [around z]), an ASC **(e)**, and an ECB **(c, e)**. The load is applied to the samples through an indenter on the tissues **(e, g)**. The carousel can contain up to 24 samples **(e)** fixed within the sample holders **(h, i)**. ASC, automatic sample changer; ECB, environment control box; MSU, mechanical stimulation unit; PRRS, physiological robot reactor system.

The PRRS is connected to a computer with a user-friendly interface to allow monitoring and controlling all system actuators, sensors, and feedback loops (Fig. 1a). The centerpiece of the device itself consists of an MSU (Fig. 1b, f, g), an automatic sample changer (ASC; Fig. 1e), and an environment control box (ECB; Fig. 1b, e). The atmosphere within the ECB is controlled with gas supplies of CO_2_, O_2_, and N_2_, all monitored and tightly operated with the interface for regulation of the individual components (Fig. 1c).

The MSU has three linear axes (orthogonal: x, y, and z axes) and one rotational axis as an additional degree of freedom (rotation around the z-axis: *R*_z_; Fig. 1f). The load (e.g., compression or torsion) generated by the MSU is transferred via an exchangeable stainless steel indenter to a sample tissue placed in a sample holder (Fig. 1g). The indenter is exchangeable and various geometries are available allowing for different contact areas and stress distribution within the sample tissue. However, only one geometry can be used within the same experimental run. The highly accurate force-feedback loops and motion systems are driven by an ultra-fast field-programmable gate array and real-time components, which continuously monitor all of the system parameters (Table 1).

**Table 1.** Technical Parameters of the Physiological Robot Reactor System That Are Defined by the Hardware and Software

Variable	Range	Accuracy
z-axis	100 mN–500 N	10 mN
Workspace (x, y, z)	150 × 22 × 40 mm	1 μm
Rotation *R*_z_	0–270°	0.1°
CO_2_	0–10%	3%
O_2_	0–25%	3%
N_2_	0–100%	NA
Temperature	Room temperature—50°C	0.1°C
Humidity	0–95%	0.3%

The DC motor, which defines the compressive force in the z-axis, can apply loads from several mM up to 500 N with an accuracy of 1 μm for a travel up to 40 mm, whereas the DC motors, defined as the shear from the xy axes, can travel up to 150 and 22 mm, respectively. The *R*_z_ axis can rotate for 270° around the z-axis. The CO_2_ concentration can be set from 0% to 10% and the O_2_ concentration up to 25% (around the ambient oxygen tension) with an accuracy of 3% of the set values. The temperature range can be set from room temperature (ambient temperature) to 50°C with an accuracy of ±0.1°C. Finally, the humidity is a passive parameter within the system, which can reach 85–95% with a variation of 0.3%.

DC, direct current.

The ASC is a rotating carousel with space for up to 24 sample holders. Each sample can be stimulated individually, that is, the type of loading pattern, the frequency, and total loading time. This allows for having extended sets of control and experimental samples in simultaneous cultures.

A custom-made sample holder made of polysulfonate material was manufactured (Amsler & Frey AG, Schinznach-Dorf, Switzerland). The holder consists of three main parts, namely, a bucket, an inlay, and a lid (Fig. 1h). Within the central hole of the inlay, a sample with a maximum diameter of 10 mm can be positioned and tightened horizontally by a stainless steel M4 screw (Fig. 1i). The volume of the culture medium in each container is 5 mL. The inlay of the sample holder has a series of bore holes and a void for a small magnetic stir bar at the bottom. Stirring is sequentially activated in the sample holders, and the design allows for efficient homogenization of the culture medium within a few seconds (Fig. 1e). The sample holder is equipped with a lid on the top to minimize both the evaporation rate of culture medium and the risk of contaminating the culture when the samples are not being subjected to mechanical loading. When the sample holder is positioned below the MSU, a lever automatically lifts the lid enabling direct contact between the indenter of the MSU and the sample (Fig. 1g).

The ECB allows for tight control of temperature, atmospheric gas composition (CO_2,_ O_2_, N_2_), and pressure (Fig. 1c). The system integrates calibrated BCP-CO_2_ and BCP-O_2_ gas sensors housing systems (Bluesens, Germany) for carbon dioxide and oxygen concentrations, respectively (Fig. 1d), which allow for continuous, accurate, and real-time *in situ* gas analysis with minimal variation (±3%) from the set values. A water tank with a ventilator, mounted on a heating element, is incorporated into the ECB (Fig. 1e) to ensure a homogenous controlled environment, in which the relative humidity reaches 85% to 95%.

### Controlling the PRRS

**FIG. 2.**
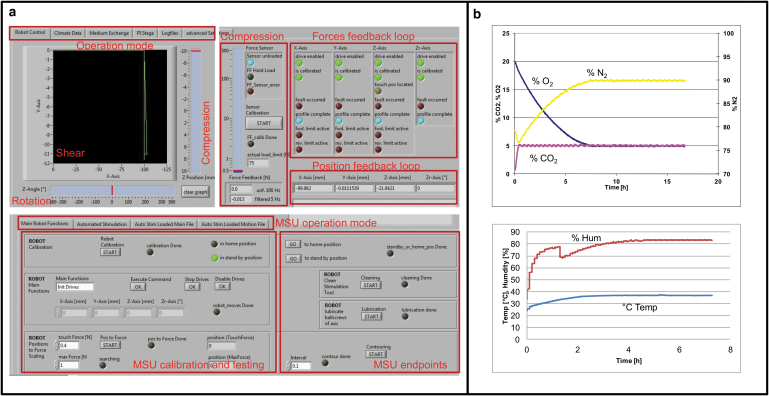
The PRRS Labview-based user interface and environmental validation feedback loops. **(a)** Screenshot of graphical user interface used to control the bioreactor system. The Labview interface enables user-defined communication and programming of individual parameters, including the mechanical loading pattern (force, frequency, and duration), environmental parameters (temperature, gas composition), and the sampling rate of the force and actuator position feedback. The individual parameters are recorded in CSV log files for control, analysis and validation of the mechanical properties of the samples, as well as the environmental parameters **(b)**. CSV, comma-separated value.

The bioreactor system is operated by customized Labview software application (National Instruments, Ennetbaden, Switzerland) allowing for both regulating the system parameters (mechanical stimulation, fluid management, and gas atmosphere), and single-automated sample manipulation (Fig. 2a and Table 1). For each sample, the stimulation cycles are programmed separately. A comma-separated value file is delivered to the software describing the loading patterns. The climate parameters, including gas composition, temperature, and humidity, were individually validated (Fig. 2b). Loads including dynamic and static compression (along z-axis), shear (sliding along x- and y-axes), and rotational movement around the z-axis (*R*_z_) within the coordinate system can be applied. The compression is force-controlled, whereas a minimal and a maximal force represent the boundary loading conditions and are set by the user. Prior stimulation, a force-distance path along the z-axis is recorded to a predefined maximal load to adjust for differences in sample geometries and nonlinear elastic properties. Each calibration is sample specific. The path corresponds to the set minimal and maximal force values and is used for calibration of the coordinate system and off-set. Within the experimental run, the indenter travels between maximal and minimal positions exerting a well-defined loading pattern, for example, dynamic compression for a predefined number of cycles and at the set frequency. However, if the measured forces during compression exceed the maximal set value or fall below the minimal, re-calibration is initiated to correct for the deviation. Recalibration may be required, for example, if creep occurs or a sample collapsed or moved along the z-axis. A similar procedure applies for static compression: The indenter moves along the z-axis to reach the maximal force and rests there for a predefined time period, unless the force deviates by a critical set value and the system readjusts for the drift or creep.

Shear forces are applied by sliding movements along the x- and/or y-axis for a predefined path distance from the center of the sample. Rotational force is applied along the z-axis for a predefined angle of rotation, frequency, and number of repetitions.

The system registers, at user-defined intervals, the position and the force experienced by the indenter. This enables the online analysis of the stiffness of the tissue during mechanical stimulation.

## Experiment

### Cell culture

Bovine articular chondrocytes were harvested from the humeral heads of 2-year-old animals obtained within 24 h post-mortem, as described earlier.^32^ The harvested cells were maintained for 48 h in a proliferation medium (Dulbecco's modified Eagle's medium [DMEM]/Ham's F12 [Gibco, Life Technologies, Zug, Switzerland] containing 10% fetal bovine serum [FBS, Sigma-Aldrich] and penicillin/streptomycin [P/S, 100 U/mL and 100 μg/mL, respectively, Gibco]), released with trypsin/EDTA, cryopreserved in 10% DMSO, and stored in liquid nitrogen. All experiments were performed with the same stock of cells. Depending on the experimental aim (Table 2), various aliquots of cryopreserved chondrocytes were seeded in monolayer culture in the proliferation medium for approximately 8 days until near confluency was reached.

**Table 2.** List of Animal Materials Used in the Experiments of the Study

Donor ID	Passage no. (population doublings)	Age (months)	Cytotoxicity	Tissue engineering	Mechanical loading/testing
B1	4 (7.2)	18	B1, B3, B4	B1, B5	B6, B7
B3	3 (10.08)	19			
B4	4 (11.43)	18			
B5	3 (9.43)	17			
B6	0 (fresh)	20			
B7	0 (fresh)	22			

The animal ID, age, passage number, and the corresponding population doubling are indicated. The number of animals with their ID and the biological replicates are indicated for each experimental purpose. For the cytotoxicity by XTT assay and tissue engineering with cell-seeded scaffolds, passaged chondrocytes (P3 and P4) were used, whereas for mechanical loading and testing fresh osteochondral explants isolated from the bovine knee joints were used.

XTT, sodium 3,3′-[1[(phenylamino)carbonyl]-3,4-tetrazolium]-bis(4-methoxy-6-nitro) benzene sulfonic acid hydrate.

### Cytotoxicity assay

An XTT (sodium 3,3′-[1[(phenylamino)carbonyl]-3,4-tetrazolium]-bis(4-methoxy-6-nitro) benzene sulfonic acid hydrate) assay (Cell Proliferation Kit II; Roche Diagnostics, Rotkreuz, Switzerland) was used to rule out potential cytotoxic effects of the complex sample holder setup versus chondrocytes. Freshly prepared cell culture medium (DMEM, 10% FBS and P/S) was filled into the sample holders of six different medium containers (*n* = 6) within the PRRS in operation and incubated for 72 h at 37°C (extraction medium). The cells were maintained in ambient atmospheric conditions (21% O_2_) supplemented with 5% CO_2_. Medium added to a 6-well cell culture plate (*n* = 6) and incubated in parallel in the ECB served as control. Subsequently, 5000 chondrocytes from passage three of three different animals were cultured for 72 h in an undiluted aliquot (100 μL) of the extraction medium in 96-well tissue culture plates. The XTT assay was performed according to the recommendations of the manufacturer.

### Scaffold cultures

Cylindrical-shaped collagen-based scaffolds were cut from the commercially available collagen membrane (Matricel, Germany; height 1.5 mm) by a sterile disposable biopsy punch (inner diameter 5 mm) and glued (Histoacryl, B. Braun Medical, Sempach, Switzerland) onto porous sintered β-tricalcium phosphate (CaP) disks (60–70% porosity; RMS Foundation, Switzerland; diameter 10 mm, height 5 mm), which served as a carrier using adhesive. The collagen-CaP scaffolds were immersed for 60 min in 5 mL DMEM to dilute/remove potentially cytotoxic substances. The pre-wetted scaffolds were seeded with 1.5 × 10^6^ chondrocytes in 30 μL differentiation medium (DMEM, P/S, ITS^+3^, 0.1 mM ascorbic acid-2-phosphate, 0.4 mM L-proline, 100 nM dexamethasone, all from Sigma-Aldrich) and cultured for up to 28 days in 24-well tissue culture plates. For the first 14 days, the cell-seeded scaffold cultures were maintained in a standard cell culture incubator to allow for maturation of the tissue-engineered constructs by supplementing the cultures with the differentiation medium containing 10 ng/mL transforming growth factor β1 (Acris Antibodies, Herford, Germany) to allow proliferation and differentiation of the chondrocytes within the constructs that were maintained in normoxic conditions (21% O_2_, 5% CO_2_) throughout the experiment. Thereafter, they were placed in the PRRS either as free swelling (unloaded controls) within 24-well plates or mounted in the sample holders for application of 5 N dynamic compression for further 14 days (1 h per day). The culture medium was changed three times a week.

### Gene expression

RNA was extracted from the cartilage explants as previously described.^33^ Briefly, the chondrocytes were released from the cartilage tissues by collagenase digestion and subjected to RNA extraction using RNeasy Mini kit (Qiagen, Basel, Switzerland) according to the manufacturer's instructions. Total RNA was reverse transcribed using murine leukemia virus (MLV) reverse transcriptase (Promega, Dübendorf, Switzerland), and quantitative PCR was performed on an ABI 7500 sequence detection system (Life Technologies) with the following Taqman Assays-on Demand: collagen type II, alpha 1 chain (COL2A1, Bt03251861_m1), collagen type I, alpha 1 chain (COL1A1, Bt03225322_m1), and aggrecan (ACAN, Bt03212186_m1).

### Histology

The collagen sponges were removed from the CaP support, fixed in 4% paraformaldehyde for 24 h at 4°C, and embedded in paraffin for subsequent preparation of 5 μm sections. The cartilage explants were removed from the subchondral bone tissues and embedded in optimal cutting temperature medium (Tissue-Tek; Haslab, Ostermundigen, Switzerland). After rehydration in graded ethanol, the sections were stained with 0.2% Safranin-O for 10 min and 0.04% Fast Green for 10 s.

### *Ex vivo* cultivation of bovine articular cartilage

Cylindrical osteochondral tissues with a diameter of 10 mm and a length of 5 to 10 mm were harvested from bovine femoral condyles of healthy 2-year-old animals, as previously described.^33^ The joints were obtained with the intact joint capsule. The osteochondral tissues were cultured in the sample holder in the PRRS for 5 days in DMEM, 10% FBS, and P/S. The culture was maintained in normoxic conditions, and the medium was refreshed every second day. The viscoelastic behavior of the articular cartilage was measured as a function of time and dynamic stimulation. The samples were subjected to sinusoidal dynamic compression (with a frequency of 0.5 Hz, maximal force of 50 N, minimal force 2 N, and deviation force-offset 20 N). The load was applied for 1 h per day for five consecutive days. All force-distance data were recorded as a function of incubation time together with the respective loading protocol information for each osteochondral tissue sample. The recorded force-displacement data were processed with Matlab (MathWorks, MA, USA) to determine the stiffness of the tissues. The contact area between the used indenter and cartilage explants was determined by ink staining, which accounted for ∼50 mm^2^, covering 80% of the total surface of the harvested cylindrical cartilage samples. The contact area is estimated for the first contact of indenter and cartilage and it increases when higher loads are applied and/or decreases when the tissue becomes stiffer. The tissue elasticity was determined based on Young's formula $E=\frac{F\times l}{\pi \times r^{2}\times \Delta l}$, where *F* is the exerted force, *l* and *r* are the thickness and radius of the cartilage, respectively, and Δ*l* is the deformation. The stiffness was determined from the equation: $k=\frac{F}{\Delta l}$.

### Statistical analysis

The quantitative XTT data obtained from three animals and six biological replicates (*n* = 3, *n* = 6) are presented with the 95% confidence interval. Statistical differences were evaluated by the Mann–Whitney non-parametric test using GraphPad Prism version 6 for windows.

The biomechanical analysis for the determination of the Young Modulus (*n* = 9) was processed with Matlab (MathWorks).

## Results

### Cytotoxicity

**FIG. 3.**
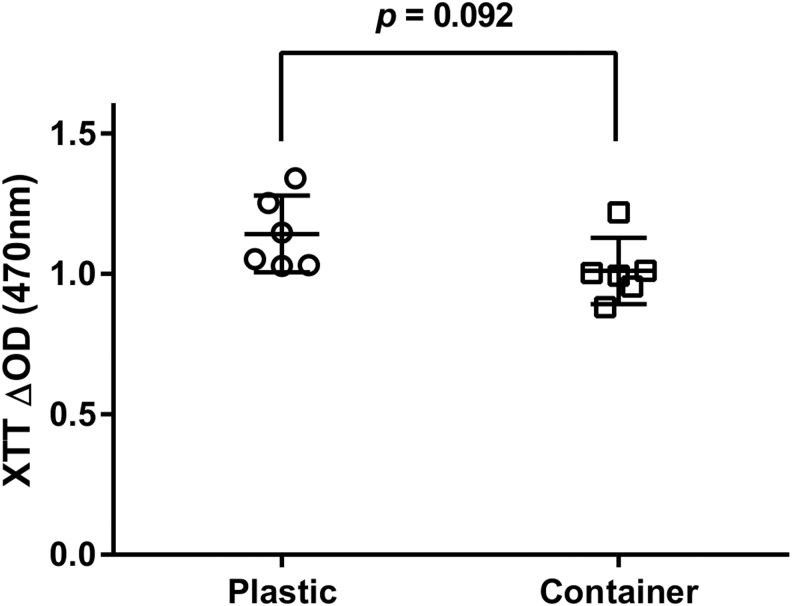
Cytotoxicity assay. Bovine articular chondrocytes from three different animals run in six biological replicates (*n* = 3, *n* = 6) were cultured for 3 days in extraction medium in either sample holders (container) or tissue culture dishes (plastic). No significant difference (*p* = 0.092) in the mitochondrial activity of the chondrocytes was found in both conditions as assayed by XTT. XTT, sodium 3,3′-[1[(phenylamino)carbonyl]-3,4-tetrazolium]-bis(4-methoxy-6-nitro) benzene sulfonic acid hydrate.

The cytotoxicity of the sample holders in the PRRS was assessed after 72 h of culture at 37°C. A reduction of 10% in XTT absorbance (*p* = 0.092) was observed in the proliferating chondrocytes as compared with the extraction medium of the standard cell culture dishes (Fig. 3). That reduction is far below the critical threshold values of 30% reduction for cytotoxicity.

### Scaffold cultures

**FIG. 4.**
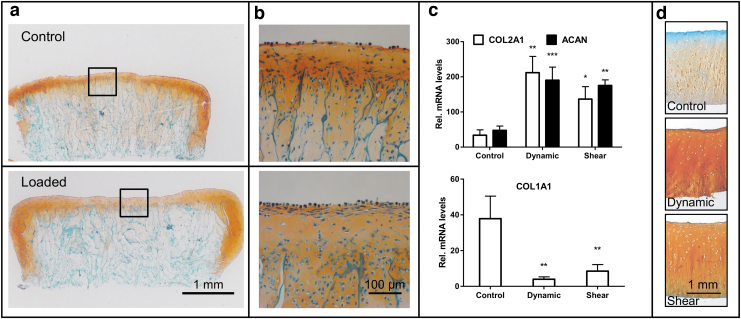
Engineering of cartilage and culture of articular cartilage explants. Chondrocytes were seeded on collagen scaffolds and cultured for 14 days in a standard cell culture incubator using differentiation medium supplemented with transforming growth factor β1. The cell-seeded scaffolds were mounted on CaP substrate and cultured for further 14 days either as controls in plastic dishes (*top figure*) or within the PRRS in the sample holders by application of 5 N dynamic compression (*bottom figure*). Histological sections of the cell-seeded scaffolds were stained with Safranin-O /Fast Green to highlight the deposition of the glycosaminoglycans. **(a)** Histological sections of the tissue-engineered cartilage tissues in control and PRRS cultures. **(b)** Depicts detailed view from regions of the cell-seeded scaffolds highlighted in *squares*. Cartilage formation and maintenance was possible in both conditions. A cartilaginous tissue with chondrocytes located in lacunae and visually a thicker cartilage layer formed in the samples cultured in the PRRS. Dynamic compression and shear stress upregulated the levels of transcripts encoding the cartilage-specific COL2 and ACAN, whereas levels of transcripts encoding COL1 were downregulated **(c)**. Moreover, dynamic compression or shear stress retained the tissue integrity, as observed histologically **(d)**.

The collagen scaffolds seeded with bovine chondrocytes were cultured either in standard plastic tissue culture dishes or within the PRRS in the sample holders by application of 5 N dynamic compression. The histological evaluation revealed that de novo cartilage tissue was engineered following mechanical stimulation in loaded samples with chondrocytes located in lacunae and visually a thicker cartilage layer as compared with the control group, which remained unchanged (Fig. 4a, b).

### Loading of cartilage explants

Fresh osteochondral explants were either subjected to a 50 N dynamic compression and/or shear stress or left as free swelling controls for 5 days. An upregulation of the cartilage-specific COL2A1 and ACAN was observed in cartilage tissues subjected to dynamic compression (*p* = 0.0015, *p* = 0.0008) or shear stress (*p* = 0.022, *p* = 0.0014), respectively, as compared with unloaded controls (Fig. 4c). In addition, a significant decrease in COL1 expression was observed in samples subjected to dynamic compression (*p* = 0.0031) and shear stress (*p* = 0.0065). Similarly, histological assessment revealed deposition of glycosaminoglycans (GAG) in loaded samples compared with almost absent GAG staining in free swelling control with a negative staining in the upper cartilage layer (Fig. 4d). Taken together, loading of cartilage samples resulted in a better maintenance and integrity of the articular cartilage tissues.

### Testing the mechanical properties of cartilage

**FIG. 5.**
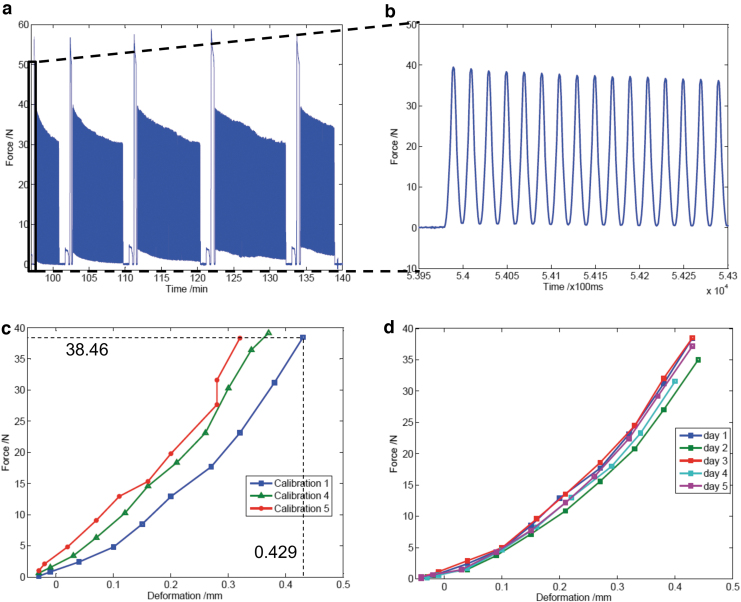
Mechanical properties of bovine articular cartilage explants. **(a, b)** The force was recorded over time during a 50 N dynamic compressive loading (**b** depicts a detailed view of **a**). **(c)** The stiffness of the cartilage was determined through consecutive calibration cycles, and **(d)** from the first calibration cycle of the daily stimulation. The diagrams of force over time, and force/deformation are shown from one representative articular cartilage tissue.

Osteochondral samples were subjected to a 5-day loading scheme with a dynamic compressive stimulation ranging from 2 to 50 N for 1 h per day for the determination of the cartilage mechanical properties. During the dynamic compressive loading, the maximal force recorded during consecutive loading cycles decreased from 50 N to less than 30 N (exceeding the force offset 20 N), necessitating further calibration steps (Fig. 5a, b). The stress-relaxation curve calculated from the repetitive calibration steps during a 1 h stimulation cycle was at the first calibration (calibration day 1) 13.4 ± 3.4 min (mean ± standard deviation) and 32 ± 1.9 min (calibration day 5) after the start of the loading (Fig. 5a). The derived Young's modulus was 3.02 ± 0.65 MPa, and it had a stiffness of 76.13 ± 16.56 N/mm. The cartilage experienced an increase in stiffness by 14%. This is also reflected in the creep behavior, which was lower at the end of the loading cycles (after 32 min) as compared with the first calibration (13 min; Fig. 5c). However, the cartilage has been found to fully recover from mechanical stress between loading cycles from day to day since the force-deformation curves are nearly identical from day 1 through day 5 (Fig. 5d).

## Discussion

Although lot of progress has been achieved toward developing clinically relevant engineered cartilage constructs, these tissues present inferior physiochemical properties as compared with native articular cartilage.^34^ The main reason is a neglect of mechanical loads in cultured cartilage constructs. It is now generally accepted that biomechanical loading stimulates the synthesis of extracellular matrix (ECM) macromolecules, which, in turn, improves the mechanical/biochemical properties of engineered cartilage constructs.^10,35,36^ Bioreactors intended for cartilage tissue engineering purposes are generally used to recreate, in part, the *in vivo* conditions so as to understand the normal cellular and molecular players and/or to mimic a pathological state of the tissue to study the pathophysiology. These bioreactor systems vary widely in their complexity and capability. Previously documented bioreactors used for the investigation of cartilage tissue engineering and cartilage explants include systems implementing hydrostatic pressure,^14,17,37^ compression,^8,38,39^ shear forces,^28,40^ and a few hybrid bioreactors implementing multiple loading regimes.^41–43^ Grad *et al.*^42^ reported a bioreactor that is able to provide dynamic or static compression and shear by oscillation surface movements through a ceramic hip ball for four stations. The system was used to investigate biaxial mechanical loading regimes using polyurethane-based scaffolds seeded with bovine articular chondrocytes on the cartilage-specific superficial zone protein and hyaluronan synthesis in atmospheric oxygen conditions. A more recent bioreactor automated system was developed to apply biaxial mechanical stimulation to engineered cartilage neotissues with the ability to maintain hypoxic conditions.^43^ The system was used to investigate the effect of uni- and biaxial loading on human articular chondrocytes encapsulated in hydrogels composed of gelatin methacryloyl and hyaluronic acid methacrylate. The study suggested that intermittent biaxial loading enhances the accumulation of cartilage-specific ECM, including ACAN and collagen type II, in hydrogel-chondrocyte loaded constructs as compared with free swelling controls.

Within the current study, we designed a user-friendly bioreactor system enabling the engineering of cartilage constructs and *ex vivo* cultivation of articular cartilage explants in an automated and well-controlled manner. We used collagen-based membranes loaded within bovine articular chondrocytes to investigate the effect of biomechanical loads. The versatility of the bioreactor system allows for investigating cell-seeded constructs and cartilage tissues under *in vivo* like conditions and improving our understanding of the mechano-transduction mechanisms of articular cartilage.

The assembled PRRS supports four degree of freedom, with compressive forces ranging from several mN to 500 N. The designed PPRS with the rotating carousel provides space for 24 sample holders, allowing for the sequential automatic application of sample-specific loading protocols. In addition, accurate control of the environmental parameters enables the generation of hypoxic conditions. Our first results obtained with the PRRS using articular cartilage were published recently.^33^ We showed previously and in this study that dynamic compression and/or shear forces applied on articular cartilage explants upregulates the levels of the transcript coding for the cartilage-specific collagen type II and ACAN, whereas a decrease in COL1 and MMP13 was observed in loaded samples as compared with free swelling controls.

In the current study, we used the collagen I/III biodegradable membrane to investigate the suitability of the bioreactor system for tissue engineering cultures. The scaffold is a white-appearing biomaterial with a rough and a smooth side. The rough side is composed of cross-linking fibers with pore sizes of ∼200 μm, and the smooth side has a compact arrangement of fibers.^44^ The collagen-based biomaterial that has an initial Young's modulus of 0.3 MPa^45^ was previously tested for tissue engineering purposes in animal cartilage defect models^46^ and is now widely used for human cartilage tissue regeneration utilizing the autologous matrix-induced chondrogenesis technique by *in vivo* implementation of human autologous chondrocytes with very satisfactory results.^47,48^

Within this study, collagen-seeded chondrocytes were successfully maintained within the PRRS for 14 days after maturation of the constructs, pointing to the suitability of the system for cell and tissue culture. In addition, the potential of the PRRS for studying the effect of high-range mechanical loading on tissue properties and maintenance of articular cartilage was demonstrated with the help of bovine cartilage explants. The measured elastic modulus was found to be 3.02 ± 0.65 MPa as initial values at day 1, which is in good agreement with the results of a previously published study that reported on the Young's modulus of human knee joint cartilage, which ranged from 2.44 to 3.66 MPa.^49^ Similarly, the detected stiffness value in our study accounted for 76.13 ± 16.56 N/mm, which is in line with previously published results on cartilage stiffness of different animals.^50^ The one hour dynamic loading cycles during the 5 day cultivation showed that the stiffness increased, which can be explained by compression of the tissue, resulting in stiffer material properties. However, within 24 h, the cartilage had fully recovered its initial mechanical properties. This emphasizes the viscoelastic behavior of cartilage, probably due to binding of the negatively charged GAG to water molecules. Our results are in good agreement with those of other studies describing the cartilage material properties.^51,52^

We used bovine articular chondrocytes and/or cartilage explants to test the suitability of the bioreactor system for tissue engineering purposes and biomechanical loading effects on cartilage formation. Further studies investigating cartilage tissue engineering using clinically relevant cells such as human autologous chondrocytes or bone marrow-derived mesenchymal stromal cells should be investigated. In addition, biomaterials with good mechanical properties should be taken into consideration. Natural biomaterials such as collagen, alginate, silk, chitosan, and fibrin present weak mechanical properties as compared with native articular cartilage and their physical properties vary widely depending on the source. Synthetic materials, such as poly(lactic-co-glycolic acid), and other polymer-based materials have the advantages to be manufactured with controllable and precise mechanical properties, including stiffness, porosity, and elasticity. These materials can be combined with a biological modifier for a better cytocompatibility and provide an enhanced adhesion and proliferation microenvironment.^53^ In addition, various biomechanical loading regimes such as shear stress superimposed on static compression with rotational movements and hypoxic conditions should be investigated.

In conclusion, the PRRS was engineered to apply a wide range of biomechanical and environmental stimuli as encountered *in vivo* within the synovial joints. The PRRS has the potential to be a convenient and flexible tool for screening and evaluation of cartilage repair strategies *in vitro*, performed under the harsh conditions encountered *in vivo* within synovial joints. The PPRS has a modular design, making it a very flexible system that can be used to stimulate sample tissues other than cartilage, such as bone and intervertebral disc with very different mechanical and environmental parameters.
